# A simple tool to evaluate the effectiveness of HIV care for settings with gaps in data availability (ESTIHIV)

**DOI:** 10.1371/journal.pone.0316794

**Published:** 2025-01-29

**Authors:** Dorthe Raben, Marie L. Jakobsen, Jamina Trajanovska, Justyna Kowalska, Anna Vassilenko, Snezana Dragas, Arjan Harxhi, Gordana Dragovic, Nadine J. Jaschinski, Bastian Neesgaard, Klaus Hjorth-Larsen, Harmony Garges, Joel Gallant, Jens D. Lundgren, Andrew Philips, Valentina Cambiano, Yazdan Yazdanpanah, Amanda Mocroft

**Affiliations:** 1 CHIP, Rigshospitalet, University of Copenhagen, Denmark; 2 University Clinic for Infectious Diseases & Febrile Conditions, Skopje, North Macedonia; 3 Medical University of Warsaw, Warsaw, Poland; 4 Belarusian State Medical University, Minsk, Belarus; 5 Clinical Center of Montenegro, Podgorica, Montenegro; 6 Faculty of Medicine, Medical University of Tirana, Albania; 7 University of Belgrade Faculty of Medicine, Department of Pharmacology, Clinical Pharmacology and Toxicology, Belgrade, Serbia; 8 ViiV. Healthcare, Research Triangle Park, North Carolina, United States of America; 9 Gilead Sciences, Foster City, California, United States of America; 10 University College London, London, United Kingdom; 11 Université Paris Diderot, Sorbonne Paris Cité, Paris, France; 12 Hôpital Bichat, Paris, France; SKYDA Health Nigeria, NIGERIA

## Abstract

Many HIV clinics with poor IT-infrastructure are unable to report data on individuals in care with HIV, on antiretroviral treatment (ART) and virologically suppressed (VS), with the aim of monitoring the HIV Continuum of Care to estimate efficacy of HIV treatment programmes. We developed an estimation-tool, ESTIHIV, and determined the minimal data required for a random sample, to produce representative estimates, with a specified level of precision, of people with HIV on ART and VS. For proof of concept, 8852 HIV positive persons from seven clinics in seven different countries, with a follow-up visit during 2017, were included. Of those, 93.8% were on ART (95% CI 93.3–94.2) and 76.7% were VS (95% CI 75.8–77.6). In 2022, we tested the tool in the RESPOND Cohort for all countries with more than 100 participants under follow-up in 2019. We included 26,426 HIV positive persons from clinics in 27 countries, 97.8% (95% CI 97.6-98.0) were on ART and 91.5% were VS (95% CI 91.2-91.8%). There was good agreement between the RESPOND country estimates of ART and VS and the estimations using a random sample calculated in ESTIHIV. With ESTIHIV, clinics can produce a reliable estimate in figures for reporting and for monitoring the effectiveness of care in their clinics.

## Introduction

Monitoring the continuum of care by assessing the number of people living with HIV receiving suppressive antiretroviral therapy (ART) is paramount for estimating the efficacy of local and national HIV treatment programmes [1]. The continuum of care for HIV outlines four stages people with HIV go through: 1^st^ stage: living with HIV, 2^nd^: being diagnosed, 3^rd^: being on ART and 4^th^: achieving viral suppression (VS) [2]. This model can help identify strengths or weaknesses in the ability to diagnose and link people with HIV to care [1–3]. According to the European Centre for Disease Prevention and Control (ECDC), 48 of 55 countries in Europe and Central Asia provided data on at least one stage of the continuum of HIV care in 2021. A total of 47 countries were able to provide data for at least two consecutive stages of the continuum (compared to 45 in 2020), and 40 countries provided data on all four stages [2,4]. This is an improvement from 2017 when the development of this project was initiated.

National level data on the two last stages of the continuum (stage 3 and 4 - people on ART and the proportion of these achieving VS) rely on both good clinical data capture and adequate reporting mechanisms between national surveillance institutions and clinical cohorts [2,5]. However, such mechanisms are not present in all countries across Europe. While many clinics have detailed information on ART use and viral dynamics, many HIV clinics have limited IT infrastructure or resources to routinely report information on all patients in care, as illustrated by the incomplete availability of continuum of care data in previous ECDC Dublin Declaration continuum of care reports [2,4,5]. Although some improvements have been observed in recent years [2], these were hampered again by the COVID19 pandemic.

Reported challenges in the past have included a lack of resources, IT-infrastructure and difficulties linking data from different sources (clinics, community centres and laboratories) to a central national surveillance centre [5]. On the other hand, prospectively collected cohort data have the advantage of linking data on a patient level. Even though a recent systematic review rated routine data from programme service delivery as being of high quality [6], high-grade continuum data were also based on methodologies that included both national surveillance data used to estimate the overall denominator for all persons living with HIV (both diagnosed and undiagnosed) and individual indicators, that can only be captured by an observational cohort design.

Nevertheless, such cohort data does not necessarily represent the entire national population. Moreover, as stated in reports of the Dublin Declaration monitoring process, many countries have difficulty acquiring patient-level data - either because of entirely lacking a functioning national HIV cohort or existing HIV cohorts not being nationally representative [1,7]

Proof of concept for the ESTIHIV tool was presented at the European AIDS Clinical Society (EACS) conference in Basel October 2019. In 2020 study activities were delayed, due to the COVID-19 pandemic. After the pandemic, the calculation method developed to calculate the sample size required for the tool, was subsequently tested in the RESPOND cohort (the International Cohort Consortium of Infectious Diseases) - a non-interventional, non-randomized, open-label, multi-cohort observational study of 19 HIV cohorts [8].

The objectives of this study were to investigate the minimal data required to make it feasible for clinics to reliably estimate the last two stages of the HIV continuum of care at the clinic and to develop a simple tool to enable clinics to calculate aggregated prevalence estimates for people on ART and with viral load (VL) suppression.

## Methodology

### Study design

The study was carried out in three parts, the first part being the proof of concept, where seven clinics provided a cross-sectional sample of data on all people with HIV under follow-up in the clinics in 2017 to calculate the proportion on ART and those virologically suppressed in each clinic.

The second part was developing the tool, using Microsoft Access. Finally, in the third part, the tool was tested using data from the RESPOND cohort.

#### Part one: Proof of concept.

Four clinics already in the RESPOND cohort were invited selected based on interest, contribution to the cohort, a high number of people living with HIV in the country and good previous collaborations. A call was sent out to clinics that previously had shown an interest in being part of the RESPOND cohort. Out of five clinics interested, three had the capacity to participate. All clinics obtained ethics approval from relevant Ethical committees as required. The four clinics already participating in RESPOND cohort, delivered data under the RESPOND approval, and the three new sites obtained approval from relevant ethics committees before entering the project.

In 2018, all seven clinics were asked to retrospectively extract data on people living with HIV seen at least once in their clinic during the period of January 1^st^ – December 31^st^, 2017 (with the option of providing data dating back to previous years). The four clinics in RESPOND were from Poland, Georgia, Belarus, and Serbia and the additional three clinics from Albania, North Macedonia, and Montenegro. We asked clinics to extract data on all people with HIV followed in their clinic for one visit during the period of January 1^st^ – December 31^st^, 2017. Inclusion criteria were all people living with HIV, ≥  18 years of age and seen at least once in the participating clinic for routine follow-up within 2017. Individuals seen multiple times during the data collection period had their most recent visit captured. Data submitted were extracted retrospectively from medical files and for this reason, consent was not required. Data were extracted on date of birth, gender, country of origin, HIV transmission group, most recent CD4 count and date of measurement, most recent VL and date of measurement, use of ART (yes/no) at most recent visit and date, date of HIV diagnosis, date of first clinic visit/registration and death. All data were anonymized and reported aggregated.

Lost-to-follow-up was not an issue as data were collected retrospectively and for only one visit. Only one country had missing data on viral suppression. The percentage of participants on ART and VS (VL < 200 copies/ml [<500 copies/ml in Belarus]) was calculated using the number in care as the denominator.

For the four clinics in RESPOND, data were submitted using the RESPOND Electronic Submission Tool (REST), a website tool that enables clinics to upload patient data electronically [9]. The software provides several quality checks of the data, allowing the clinics to correct potential errors before the final data upload. The three new clinics submitted data using REDCap, and data were collected and managed using REDCap electronic data capture tools hosted at CHIP, Rigshospitalet, University of Copenhagen, Denmark [10,11]. REDCap (Research Electronic Data Capture) is a secure, web-based software platform designed to support data capture for research studies, providing 1) an intuitive interface for validated data capture; 2) audit trails for tracking data manipulation and export procedures; 3) automated export procedures for seamless data downloads to common statistical packages; and 4) procedures for data integration and interoperability with external sources. All data for the proof of concept were submitted by July 30^th^, 2018, and database was closed.

#### Clinic survey.

A clinic survey was circulated to all seven participating clinics to collect basic background data on HIV clinical care and treatment at clinical and country level through a brief online survey in REDCap. Questions addressed issues at the national and clinic level on HIV treatment and care (supporting information 1). The principal investigators at the participating clinics/sites completed the survey. Data reported by the clinics were compared to the country level data the same countries reported in 2018 to the UNAIDS Data report [12]. The aim was to assess how representative each clinic was compared to all HIV clinics within the country.

#### Part two: Developing the tool – ESTIHIV, Estimation for HIV treatment and viral suppression.

A calculation formula [13] for a random sample size was developed in Excel, based on clinic population and the estimated level of VS, to provide the number of people with HIV in a given clinic population required for a random sample to be representative of the entire clinic population [14].

The formula used calculates a sample size for large populations. This formula estimates the sample required to allow a 95% confidence interval of ±  e% if the proportion of patients with virologic suppression is p%.


$$\text{n}0=3.84*\text{p}*\left(1–\text{p}\right)/{\text{e}}^{2}$$

p = the proportion with virologic suppression within the clinic.

e = precision, i.e., required precision of the confidence interval and adjusted for the finite population correction factor as


$$\text{n = n0/ }\left(\text{1 + }\left(\left(\text{n0–1}\right)\text{/N}\right)\right)$$

Where N is the size of the clinic population.

We used Microsoft Access to develop ESTIHIV. A downloadable standardised instrument to create an electronic patient database for clinics with large patient populations under follow up, and limited IT resources.

#### Part three: application of the tool in the RESPOND Cohort.

In 2022, for the purpose of testing the tool, data from all countries within the RESPOND HIV cohort were included for comparability purposes, with each country treated as a separate population to calculate the number under follow-up, on ART and with VS. Thus, for each country participating in RESPOND with > 100 persons under follow-up during 2019, we calculated the percentage on ART and the percentage with VS (<200 copies/ml or < limit of detection if limit of detection ≥  200 copies/ml) at the last visit in the prior 12 months. All assessments used the number of people under follow-up (defined as seen in a clinic at least once during the last 12 months) as the denominator. We used the tool to calculate the required sample size (*required n*) from each country-specific population needed to estimate the number of people on ART and the number virologically supressed based on the estimated size of the population, the desired width of the confidence interval (CI) for the estimate, set at 5%, and the estimate of the percentage of people with HIV on ART and VS obtained from the total country-specific population [14]. A random sample of *required n* individuals was selected within each country-specific population using a random number generator with a uniform distribution. The percentage receiving ART and with VS from the random sample was compared to those not randomly selected to test the tool and random sampling techniques. Differences were assessed using the chi-squared and/or Fishers exact test. Sensitivity analyses investigated the impact of using a fixed estimate of 71%, 80% or 90% for each country-specific population rather than the derived estimates.

All data were analysed using Statistical Analysis Software version 9.4 (Cary, NC, USA).

## Results

### Proof of concept

**Fig 1:**
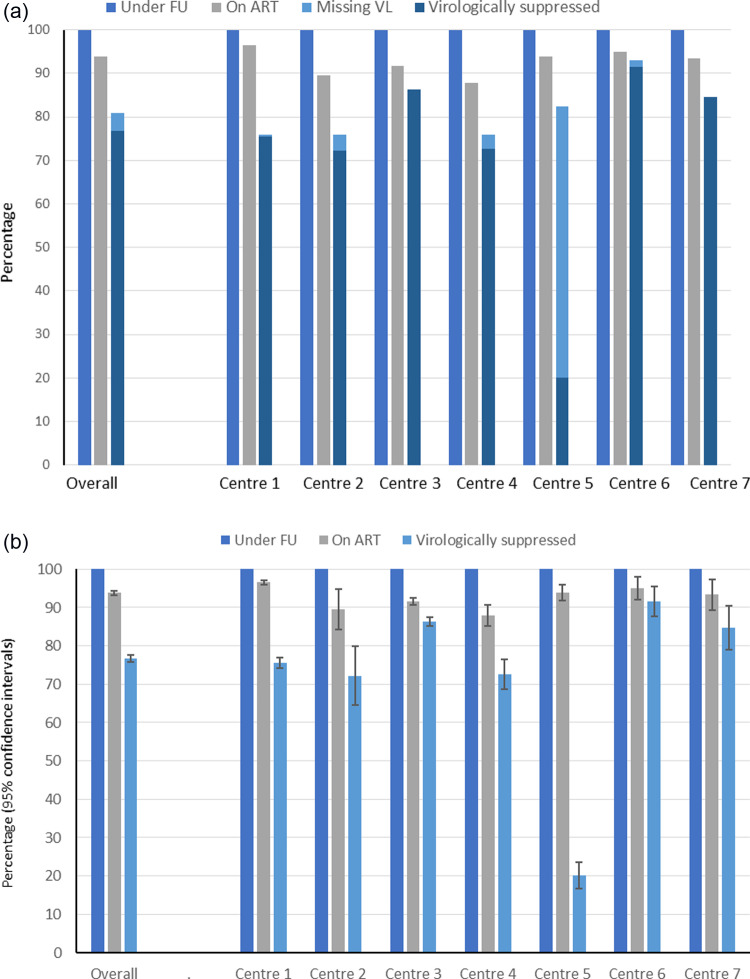
a) Continuum of care for the participating 7 clinics (anonymised) for the proof of concept in 2017 on: a. missing viral load; b) % on ART and VS at last clinic visit.

For the proof of concept, we collected data from 8852 persons: Georgia (n = 3839), Albania (n = 542), North Macedonia (n = 202), Montenegro (n = 150), Serbia (n = 521), Belarus (n = 133) and Poland (n = 3465). Median age was 40 (interquartile range [IQR] 34–48) and median CD4 count was 548 (IQR 360-753/mm^3^); Table 1. Overall, 93.8% were on ART (95% CI 93.3–94.2) and 76.7% were VS (95% CI 75.8–77.6%), where individuals without a viral load measurement were assumed not to be VS. There were considerable differences in ART and VS across clinics, and in the percentage with missing information on VS in Fig 1a, leading to a lower percentage of individuals with VS - Fig 1b.

**Table 1:** Characteristics of the proof of concept population vs. RESPOND Cohort population.

		Proof of concept population		RESPOND population	
		N	%	n	%
**All**		**8852**	**100**	**26426**	**100**
**Age**	≤30	1255	14.2	944	3.6
	30–40	3046	34.4	4017	15.2
	>40	4550	51.4	21465	81.2
	Missing	1	0.0	0	0
**Last CD4**	≤500	3833	43.3	6617	25.0
	>500	4900	55.4	17702	67.0
	Missing^*^	119	1.3	2107	8.0
**Gender/ risk of HIV acquisition**	MSM	3157	35.7	12119	45.9
	Male heterosexual	1470	16.6	4173	15.8
	Female heterosexual	1544	17.4	5115	19.4
	Male IDU	1648	18.6	2245	8.5
	Female IDU	197	2.2	938	3.6
	Male other	669	7.6	1143	4.3
	Female other	165	1.9	635	2.4
	Non-Male/-Female	0	0	58	0.2
**Years since HIV diagnosis**	≤1	1180	13.3	0	0
	1–3	1922	21.7	0	0
	>3	5719	64.6	24254	91.8
	Unknown	31	0.4	534	2.0

* In 12 months before last visit.

### Clinic survey

Results of the clinic survey are presented in Table 2. For comparison, reported country level data is shown for the contributing countries/clinics as reported by UNAIDS [4]. The data reported shows great variation in size of clinic populations across countries, as well as number of clinics in each country. For Albania, Georgia, Montenegro, and North Macedonia almost all people under follow-up in the country, were contributing data to the analysis, making the reported data representative not only at the clinic, but also at the country level.

**Table 2:** Clinic survey on National and clinic data on HIV treatment and care, 2018 compared to data reported to UNAIDS at the same time period.

	Country Level					Clinic Level					
	Unaids Report 2019					Clinic Survey Data			Mode of transmission % (estimated)		
Country	People with HIV	People with HIV - status known	People with HIV under follow-up	MSM/IDU	Clinics providing HIV care	People with HIV under follow-up	People with HIV on ART	Contribution to ESTIHIV	MSM	IDU	Heterosexual
**Albania**	NA	NA	580	NA/NA	< 3	501–1000	424–850 (85%)	545	27–82 (5–15%)	< 27 ( < 5%)	> 272 ( > 50%)
**Belarus**	27000 (22–34000)	21330 (79%)	15930 (59%)	NA/10.935 (40.5%)	> 30	> 2000	> 1080 (54%)	153	< 8 ( < 5%)	46–77 (30–50%)	<77 (<50%)
**Georgia**	9400 (8100–11000)	5546 (59%)	4606 (49%)	NA/NA	4–10	> 2000	>1700 (85%)	4437	444–666 (5–15%)	1331–2219 (30–50%)	1331–2219 (30–50%)
**Montenegro**	< 500	< 275 (55%)	< 200 (40%)	NA/NA	< 3	< 250	< 225 (90%)	150	<75 (> 50%)	< 7 ( < 5%)	23 – 45 (15–30%)
**Poland**	NA	NA	NA	(68.3%)/ (46.2%)	16–20	> 2000	>1900 (95%)	4122	2061 ( > 50%)	206–618 (5–15%)	206–618 (5–15%)
**N. Macedonia**	< 500	295 (59%)	270 (54%)	NA/NA	< 3	< 250	225–238 (90–95%)	202	<101 (> 50%)	0	30–61 (15–30%)
**Serbia**	3000 (2200–3800)	2580 (86%)	1950 (65%)	NA/NA	4–10	1000–1500	1200 (80%)	926	NA	NA	NA

### Tool

When the ESTIHIV tool is downloaded, the user creates a personal log in and password. A user guide is available (supporting information 2). The first entry allows users to enter data on the clinic.

The next step is to ‘Choose data entry method’ depending on the size of the clinic patient population, the user can choose between entering all patients, relevant in clinics with a small population under follow-up, or creating a random sample - for clinics with a larger population. The user can also choose how accurate the estimate of VS level should be: very high, high or moderate: the higher the accuracy, the larger the sample.

With this information, the tool calculates how many patients are needed for a random sample and an estimation of the hours of work needed for data entry, based on an estimate of 15 minutes per patient. A function within the tool provides a guide for the user on how to ensure a sample is randomly selected. (Supporting information 2). A random sample is important to avoid selection-bias. Patient can be files ordered in different ways, i.e., alphabetically, by date of birth, by region or an entirely different way. Based on this every 5^th^/10^th^/20^th^/-- patient can be selected, until the number needed for the random sample is reached. Example: total HIV patient population in a clinic: 200 patients and sample needed: 25, in this case clinic can pick every 8^th^ patient until 25.

Patients are entered with one form per patient entry. For each data field in the entry form, there is an information function that guides the user in entering data correctly. Data quality checks are built in to ensure dates are genuine and drop-down options are made for gender and mode of transmission. The complete database can be accessed via ‘edit patient data’ for editing in the database. After data have been entered, they can be exported to an overview of the patient population on a graph – ‘Clinic Continuum of Care’ - in pdf or Excel format.

### Application of the tool in the RESPOND HIV cohort

**Fig 2:**
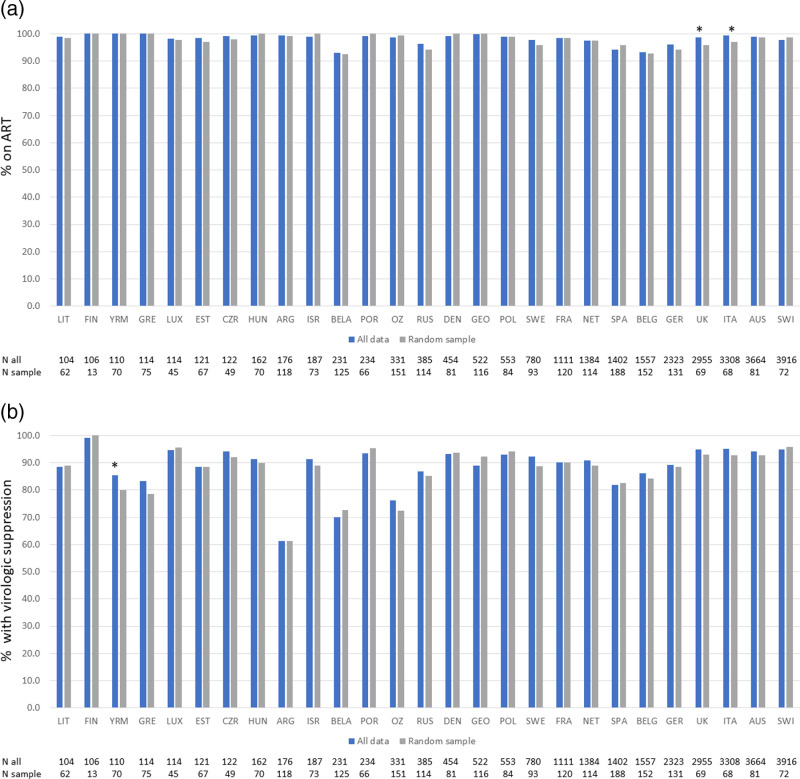
Application and testing of the tool to RESPOND Cohorts: a) comparison of all data vs random samples on ART and b) comparison of all versus random sample on virologic suppression** in RESPOND country cohorts, year 2019. *p < 0.05. **Missing assumed failure.

A total of 27 clinics in the RESPOND HIV cohort that had > 100 individuals in follow-up during 2019 and contributed data to the testing of the tool – part three. Overall, 26,426 persons were included; their characteristics at last visit are shown in Table 1. They differ markedly from the proof-of-concept population; they are older, with higher CD4 counts, a higher percentage of MSM and have been diagnosed with HIV longer. Overall, 97.8% were on ART (95% CI 97.6-98.0); ranging from 93.1% to 100% within country-specific populations, as shown in Fig 2a. In total, 91.5% were VS (95% CI 91.2-91.8%), with the small percentage with missing data (3.5%) classified as not suppressed. Most country-specific populations had VS in excess of 90%, Fig 2b; the lowest rates of VS were 70.1%, and at least partly explained by 24/231 (10.4%) of the country-specific population having no data on VL available, and therefore included as unsuppressed.

Using the tool and the randomly selected individuals from each country-specific population, the full continuum of care was compared to the continuum of care in the random sample in Fig 2a and 2b. For example, there were 104 individuals from the first country-specific population included in the testing exercise. Based on the observed virologic suppression rate of 88.5%, and a required 5% limit for the confidence interval, the tool recommended a random sample of 63 individuals. Among this random sample of 63 individuals, the percentage receiving ART was similar to the total country-specific population (99.0 versus 98.4% respectively, p = 0.42), as was the percentage virologically suppressed (88.5 versus 88.9% respectively; p = 0.87). There was only one country-specific sample population where the percentage with virologic suppression, 80.0%, differed significantly from the total country-specific population, 85.5%, p = 0.032. Each country from the included RESPOND cohort had an individual % on ART and VS as shown in Fig 2a and 2b. These country-specific estimates were applied to the mathematical formula used to estimate the number needed to produce a reliable estimate, based on the required level of precision [14]. The derived estimate varies between countries as shown in Fig 2a and 2b.

Results from sensitivity analyses which assumed a different (fixed at 71%, 80% and 90%) level of VS within each country population showed highly consistent results. The most notable differences were the size of the random sample, where lower rates of virologic suppression required a higher number for the random sample.

## Discussion

Seven clinics in RESPOND provided data for testing ‘proof of concept’ and constructing the two last stages of the of the HIV continuum of care. We developed a tool – ESTIHIV - that provides the sample size required to estimate the two last stages of the HIV continuum, so that clinics with more limited IT resources can set-up an electronic version of a subset of their data. When applying ESTIHIV to an existing HIV Cohort there was good comparability regarding the two last stages of the continuum – ART and VS - in the cohort and the estimations calculated using a random sample.

We provide proof of concept that collecting data from a small random sample of a clinic population of people living with HIV, can produce a reliable estimate of the two last stages of the HIV continuum, which means that clinics can produce a reliable continuum of care without the need to have all patient level data collected or available for analysis for all people in care. This methodology was developed into a simple and user-friendly downloadable tool – ESTIHIV – enabling clinics to estimate these Figs for reporting and for a self-applied auditing tool, strengthening monitoring of the effectiveness of care in different population subgroups.

The ESTIHIV tool can fill a gap in reporting to European institutions like ECDC by allowing clinics to estimate the proportion of people on ART and VS. The tool has the potential to support not only surveillance reporting but also to measure progress over time. Also, it allows selection of a specific risk group and to measure ART and VS in this particular group. This could be particularly interesting, as data in certain risk groups are often lacking [6], and there is a particular interest in measuring progress in treatment uptake in sub-population groups. The tool can also be used outside of Europe in resource limited settings.

The application of the sample size calculation formula showed good agreement between the full country specific data and the random sample. We found 1 of 27 country-specific populations where the percentage virologically suppressed using the tool and a random sample differed significantly compared to using all the individuals in the clinics. One would expect 1 in 20 of the p-values comparing the sample to be < 0.05 through chance alone, which is in line with that error rate. In each of three separate sensitivity analyses using a fixed rate of virologic suppression of 71%, 80% or 90%, and identifying a different random sample from each of the country-specific populations, one of the random samples differed significantly from the country-specific populations, and 26 were not statistically different. In each sensitivity analysis, a different country-specific random sample differed from the whole population, which is also in line with the type I error rate of 5%.

### Study limitations

This study has several limitations. The countries selected for the proof-of-concept, part 1, of this study were not randomly selected. Therefore, they may not be representative of countries that cannot provide data for the continuum of care. Furthermore, we used the number in care as denominator for calculating the percentage of viral suppression. One of the countries had a large amount of missing data for VS, and assuming these individuals are not virologically suppressed will likely underestimate virologic suppression in this country. We used the RESPOND cohort to test the tool, using each clinic as a population, but the individuals from each country enrolled in RESPOND are likely a selected sample of all people living with HIV in any country. However, in smaller countries with fewer clinics, the results are close to the country population as presented in Table 2, as the clinics that contributed to RESPOND care for a large proportion of the population in care in that country. The tool also relies on identifying a random sample, which might be challenging when records are not computerized or for countries with scarce resources.

It is also worth noting that we have presented ESTIHIV using the number in care for the denominator for both ART and VS, in contrast to UNAIDS 95-95-95, where the previous bar number diagnosed and number on ART are used as denominators for ART coverage and VS, respectively. Future development of the tool could allow estimates of people living with HIV on ART and VS to be presented both ways.

### Strengths

The study also has several strengths. The simple tool allows clinic or country-level populations to estimate the continuum of care using a random sample of the people with HIV in follow-up. Furthermore, the tool’s calculation method was tested on a different population and showed few differences between the actual clinic continuum and the random sample. The tool has the potential to support generation of continuum of care data for different sub-population groups, including key populations at higher risk of HIV acquisition such as men who have sex with men, people who inject drugs, transgender populations, and others.

## Conclusion

To conclude, the ESTIHIV tool has the potential to support clinics in estimating clinic-specific percentages of people living with HIV on ART and VS for quality control and benchmarking, as a self-applied auditing tool as well as for National and European reporting purposes, increasing the availability of data in countries with otherwise incomplete national level data and fragmented data on VS. The continuum of care is an important instrument to monitor progress towards reaching the Sustainable Development Goals and monitoring the effect of local and national treatment programmes. To construct the continuum of care necessitates good data which is not always available on country level or even clinic level. The ESTIHIV tool provides a solution to this challenge. Finally, ESTIHIV can be used for generation of continuum of care data by sub-populations and could be modified and used for collection of data on other infections, such as hepatitis B and C.

## Supporting information

S1 File ESTIHIV clinic survey. (PDF)

S2 File ESTIHIV user guide. (PDF)
