# Supplementary material for: A simple tool to evaluate the effectiveness of HIV care for settings with gaps in data availability (ESTIHIV)
Source: PLoS One. 2025 Jan 29;20(1):e0316794. doi: 10.1371/journal.pone.0316794 (PMC11778770; doi:10.1371/journal.pone.0316794)
Supplement: S1 File — (PDF) [file pone.0316794.s001.pdf]

RESPOND PH, Proof of Concept - clinic survey

Record ID	______________________
Name:	______________________
Country:	______________________
RESPOND site number:	______________________
NATIONAL HIV CARE	
How many HIV clinics are there in your country for adult HIV care? (estimated)	◯ < 3<br>◯ 4 - 10<br>◯ 11 - 15<br>◯ 16 - 20<br>◯ 21 - 30<br>◯ > 30
Are there specialised clinics for HIV care for the following populations:	☐ Children (Pediatric HIV)<br>☐ People in prisons<br>☐ Other<br>☐ No
If other, please describe:	______________________
Do you have National HIV treatment guidelines?	◯ Yes<br>◯ No
If no, which guidelines do you use?	◯ EACS - European AIDS Clinical Society - guidelines, version 9.0 - October 2017<br>◯ WHO - World Health Organisation - treatment guidelines, 2010 revision.<br>◯ CDC - Centre for Disease Control - Guide for HIV/AIDS clinical care, revision 2014<br>◯ Other
Please enter name and version of guidelines used:	______________________
Is HIV care available and accessible in the most affected areas in your country?	◯ Yes<br>◯ No
Please provide more information:	______________________
CLINIC INFORMATION	

What is the total number of doctors - Full Time Equivalent*, FTE - involved in HIV care, working at your clinic?	◯ < 5<br>◯ 5 - 10<br>◯ > 10<br>((*1 FTE = 1 full time employed doctor or 2 part time doctors))
What is the total number of nurses - Full Time Equivalent*, FTE - involved in HIV care, working in your clinic?	◯ < 5<br>◯ 5 - 10<br>◯ 11 - 15<br>◯ 16 - 20<br>◯ > 20<br>((*1 FTE = 1 full time employed nurse or 2 part time nurses))
How many HIV positive patients does one doctor - one FTE* - see in a week?	◯ < 10<br>◯ 10 - 20<br>◯ 21 - 30<br>◯ 31 - 40<br>◯ 41 - 50<br>◯ > 50<br>((*1 FTE = 1 full time employed doctor or 2 part time doctors))
Comments to the above question:	________________________________
If possible, is the patient seen by the same doctor at each follow-up visit?	◯ Yes, it is always possible<br>◯ Yes, ideally - but not alway possible<br>◯ No, not possible<br>◯ Not a priority
Do you have an Electronic Patient Medical Record or equivalent?	◯ Yes<br>◯ No
Is all HIV treatment related information on the patient captured in this Electronic Patient Medical Record or equivalent?	◯ Yes<br>◯ No
If no, what HIV treatment related data is NOT captured electronically?	________________________________
Can HIV positive patients receive HIV care in other clinics besides HIV clinics - i.e. their GP?	◯ Yes<br>◯ No
If yes, please describe what other kind of clinics provide HIV care?	________________________________
How often is viral load meassured in stable HIV patients?	◯ < than every 3 months<br>◯ every 3 months<br>◯ every 6 months<br>◯ every 12 months<br>◯ > every 12 months
Do you get notified when an HIV patient moves to another clinc for HIV care?	◯ Yes<br>◯ No
Do you get notified when an HIV patient in your Clinic leaves the country/ emigrates?	◯ Yes<br>◯ No

How do you get notified when a patient in your clinic dies?	☐ by relatives<br>☐ by other clinics/ hospitals<br>☐ by national registry<br>☐ my clinic does not get notified
PATIENT POPULATION	
What is the total number of HIV positive patients under follow-up in your clinic?	○ < 100<br>○ 101 - 200<br>○ 201 - 300<br>○ 301 - 400<br>○ 401 - 500<br>○ > 500
What percentage of HIV positive patients seen for follow-up in your clinic, receive ART?	______________________
What is the estimated % of patients in the below mentioned transmission groups, seen in your clinic?	
Homo/ bi-sexual men:	○ 0<br>○ < 5 %<br>○ 5 - 15%<br>○ 15 - 30%<br>○ 30 - 50%<br>○ > 50%<br>○ Do not know
Injecting drug users:	○ 0<br>○ < 5 %<br>○ 5 - 15%<br>○ 15 - 30%<br>○ 30 - 50%<br>○ > 50%<br>○ Do not know
Homo/bi-sexual man + inj drug user:	○ 0<br>○ < 5 %<br>○ 5 - 15%<br>○ 15 - 30%<br>○ 30 - 50%<br>○ > 50%<br>○ Do not knoww
Vertically infected:	○ 0<br>○ < 5 %<br>○ 5 - 15%<br>○ 15 - 30%<br>○ 30 - 50%<br>○ > 50%<br>○ Do not know
Heterosexual contact:	○ 0<br>○ < 5 %<br>○ 5 - 15%<br>○ 15 - 30%<br>○ 30 - 50%<br>○ > 50%<br>○ Do not know

Sex-worker:	○ 0<br>○ < 5 %<br>○ 5 - 15%<br>○ 15 - 30%<br>○ 30 - 50%<br>○ > 50%<br>○ Do not know
Other:	○ 0<br>○ < 5 %<br>○ 5 - 15%<br>○ 15 - 30%<br>○ 30 - 50%<br>○ > 50%<br>○ Do not know
Unkown:	○ 0<br>○ < 5 %<br>○ 5 - 15%<br>○ 15 - 30%<br>○ 30 - 50%<br>○ > 50%<br>○ Do not know
