# Supplementary material for: A simple tool to evaluate the effectiveness of HIV care for settings with gaps in data availability (ESTIHIV)
Source: PLoS One. 2025 Jan 29;20(1):e0316794. doi: 10.1371/journal.pone.0316794 (PMC11778770; doi:10.1371/journal.pone.0316794)
Supplement: S2 File — (PDF) [file pone.0316794.s002.pdf]

Supporting documentation 2 - ESTHIV user instructions

ESTIHIV – Estimation tool for HIV Treatment and Viral Suppression

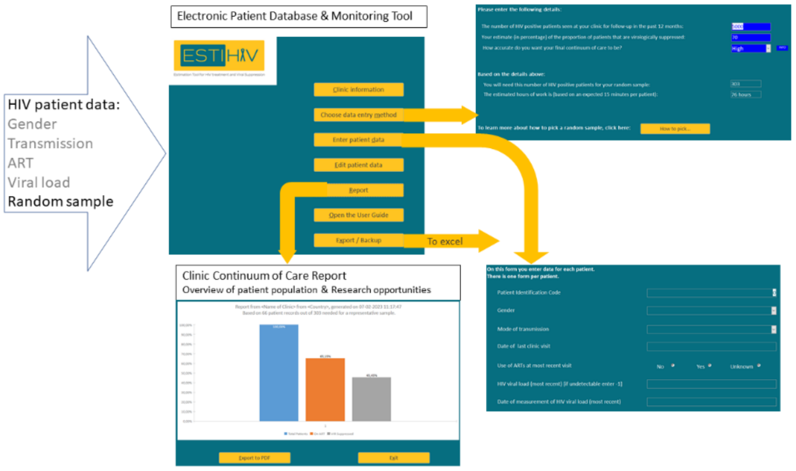

With this tool it is possible to calculate an estimated 'right-hand side' of the Continuum of Care for people with HIV followed up in your clinic - the proportion in treatment and proportion virologically suppressed (VS). This will help you reach the ambitious but achievable 90-90-90 goals, set by UNAIDS 2015.

The tool is accessible here*: download the ESTIHIV TOOL. If you have any questions, please write to respond.rigshospitalet@regionh.dk subject: **ESTIHIV**

ESTIHIV has been developed based on results from a research project under the RESPOND Consortium that investigated the minimal data required to estimate the right-hand side of the continuum.

ESTIHIV calculates the 'right-hand side' of the HIV continuum of care; percentage of patients in the clinic on ART and the percentage of patients who are virologically suppressed, by uploading patient data on a random sample, calculated by the tool, of people with HIV followed-up in a clinic. Results are delivered in a report showing the 'right-hand side' continuum for the clinic at the given point in time.

Get started:

1. Start by creating a log-in. You can use the default user "User" to log in or appoint an Administrator to set up multiple users. Please note however, that all users have access to the same data, so only one user at the time can access.

2. Go to **'Clinic Information'** and enter your clinic information. This only has to be done once and will appear on the final report

3. Go to **'Choose your method of data entry'** – here you have two options:

    a. **I will use data on all patients:** go to *Enter patient data* and start entering data on all patients seen for follow-up in your clinic within the past 12 months. This can be useful if your clinic has few people with HIV under follow-up (less than 100 +/-)

b. **I will use a random sample:** When choosing a random sample, there are important aspects to consider.

Please read the instructions carefully in the sample calculation section and have the following information available:

1. The total number of people with HIV seen at your clinic for follow-up in the past 12 months.
2. Your estimated proportion, in percentage, of the number of above, who are virologically suppressed.
3. Choose how accurate you want your final continuum to be in the drop-down function. This information is used to estimate the size of the 95% confidence interval (CI) for your continuum:
   I. **Very high** - corresponding to 1%. If you estimate that 81% of your population is virologically suppressed, the lower limit of the estimated 95% confidence interval will be 80% and the upper limit will be 82%.
   II. **High** – corresponding to 5%. If you estimate that 81% of your population is virologically suppressed, the lower limit of the estimated 95% confidence interval will be 76% and the upper limit will be 86%
   III. **Moderate** – corresponding to 10%. If you estimate that 81% of your population is virologically suppressed, the lower limit of the estimated 95% confidence interval will be 71% and the upper limit will be 91%

With the above information the tool will calculate *the number needed for your random sample* and *the estimated working hours needed* for entering the sample in the database.

4. **How to make the sample random** is explained in more details in the sample calculation section under the function ***How to pick a random sample****:*

A random sample is important to avoid bias when selecting patients. You may have ordered your patient files alphabetically, by date of birth, by region or an entirely different way. Based on that you pick every 5th/10th/20th/-- patients, until you reach the number needed for your random sample. Example: total HIV patient population: 200 and sample needed: 25. Please pick every 8th patient until you reach 25 patients.

5. Go to **Enter patient data** and enter the number of patients needed for your sample and make sure patients are randomly picked.

Patient data required:

a. Internal patient identifier code
b. Gender
c. Mode of transmission
d. Date of last clinic visit
e. Use of ART at most recent visit (yes/no)

   f. HIV viral load – most recent

   g. Date of measurement of HIV viral load – most recent

6. Keep a patient identification list. You can choose a patient coding system, without using recognizable patient data or let the system generate one for you. Please use numbers only.
7. If you find a mistake in the dataset, go to **Edit patient data** and click on the relevant patient entry. Choose to **edit** or **delete,** depending on what is needed.
8. Go to **Report** to see your clinic continuum of care.
9. If you have any questions or feedback, please write to: respond.rigshospitalet@regionh.dk subject: **ESTIHIV.**
